# A novel approach for joint line restoration in revision total ankle arthroplasty based on the three-dimensional registration of the contralateral tibia and fibula

**DOI:** 10.1186/s40634-023-00579-y

**Published:** 2023-02-03

**Authors:** S. Hodel, A. K. Calek, N. Cavalcanti, S. F. Fucentese, L. Vlachopoulos, A. Viehöfer, S. H. Wirth

**Affiliations:** Department of Orthopedics, Balgrist University Hospital, University of Zürich, Forchstrasse 340, CH-8008 Zürich, Switzerland

**Keywords:** Total ankle arthroplasty, Revision total ankle arthroplasty, Joint line

## Abstract

**Purpose:**

The use of total ankle arthroplasty (TAA) is increasing over time, as so will the need for revision TAAs in the future. Restoration of the ankle joint line (JL) in revision TAA is often difficult due to severe bone loss. This study analyzed the accuracy of a three-dimensional (3D) registration of the contralateral tibia and fibula to restore the ankle joint line (JL) and reported side-to-side differences of anatomical landmarks.

**Methods:**

3D triangular surface models of 96 paired lower legs underwent a surface registration algorithm for superimposition of the mirrored contralateral lower leg onto the original lower leg to approximate the original ankle JL using a proximal, middle and distal segment. Distances of the distal fibular tip, anterior and posterior medial colliculus to the JL were measured and absolute side-to-side differences reported. Anterior lateral distal tibial angle (ADTA) and lateral distal tibial angle (LDTA) were measured.

**Results:**

Mean JL approximation was most accurate for the distal segment (0.1 ± 1.4 mm (range: -3.4 to 2.8 mm)) and middle segment (0.1 ± 1.2 mm (range: -2.8 to 2.5 mm)) compared to the proximal segment (-0.2 ± 1.6 mm (range: -3.0 to 4.9 mm)) (*p* = 0.007). Distance of the distal fibular tip, the anterior, and posterior medial colliculus to the JL, ADTA and LDTA yielded no significant side-to-side differences (n.s.).

**Conclusion:**

3D registration of the contralateral tibia and fibula reliably approximated the original ankle JL. The contralateral distal fibular tip, anterior and posterior medial colliculi, ADTA and LDTA can be used reliably for the planning of revision TAA with small side-to-side differences reported.

**Level of Evidence:**

IV.

## Introduction

Osteoarthritis of the ankle joint accounts for approximately 1% of all forms of osteoarthritis in the adult population [25]. The literature indicates a post-traumatic genesis in up to 80% [4, 18]. While arthrodesis of end-stage osteoarthritis of the foot represents a widely used treatment, impaired functional outcome after ankle fusion has been reported in the long-term due to the loss of hindfoot motion and the risk of secondary adjacent joint arthritis [11, 17]. Total ankle arthroplasty (TAA) on the other hand allows preserving range of motion and possibly prevents degeneration of adjacent joints and led to a tremendous boom of TAA in recent years [12, 23].

Despite reliable outcomes after TAA for ankle arthritis, there is a paucity of literature regarding the restoration of the native joint line (JL) and whether changes in JL level affect clinical outcome. Given the increasing aging of the population, a rising number of primary TAA cases are expected in the upcoming years, followed by revision cases [21].

In a previous study, Harnroongroj et al. [8] demonstrated an increased ankle JL in end-stage osteoarthritis compared to the healthy side. The joint line height ratio based on contralateral weight-bearing radiographs was used for their measurements. However, a major limitation of this method is that it depends on distal tibial, fibular, and talar bony landmarks, which may not be preserved after trauma or in a revision situation and therefore are of limited value.

Additional proximal landmarks would be of great help in approximating the JL and could improve the accuracy of ankle anatomy reconstruction as previously demonstrated [5]. Measurement techniques based on CT-reconstructed three-dimensional (3D) models of bone anatomy using a mirrored model of the contralateral bone anatomy as a template for the normal anatomy have demonstrated excellent accuracy in previous studies [9, 24]. To the best of our knowledge, there is no method for ankle JL restoration based on a contralateral 3D registration [8].

The primary aim of this study was to evaluate the accuracy of a novel 3D registration algorithm based on the contralateral side to restore the JL involving defined segments of the tibia and fibula for registration. The secondary aim was to analyze side-to-side differences of currently used anatomical landmarks in TAA and to analyze their influence on the accuracy of JL restoration. We hypothesized (1) that the JL could be accurately approximated based on contralateral 3D registration incorporating the tibia and fibula as anatomical landmarks and (2) that no relevant side-to-side differences exist that would influence the accuracy of JL approximation.

## Materials and methods

### Study cohort

Ninety-six cadaveric specimens of the lower leg without previous trauma, surgery or deformity, were provided by the Institute of Forensic Medicine, University of Zurich and included for analysis. Thirty-four male and twelve female donors (missing gender information in two specimens) with a mean age of 52 years ± 17.7 (range: 21 to 95 years) were assessed. The mean weight was 83.1 ± 16.5 kg (range: 55 to 111 kg) and the mean height was 176.2 ± 8.6 cm (range: 154 to 195 cm).

The Ethics Committee Zurich approved the acquisition of the computer-tomography data by the Institute of Forensic Medicine, University of Zurich. All data were provided anonymously.

### Computer-tomography examination and three-dimensional registration

**Fig. 1:**
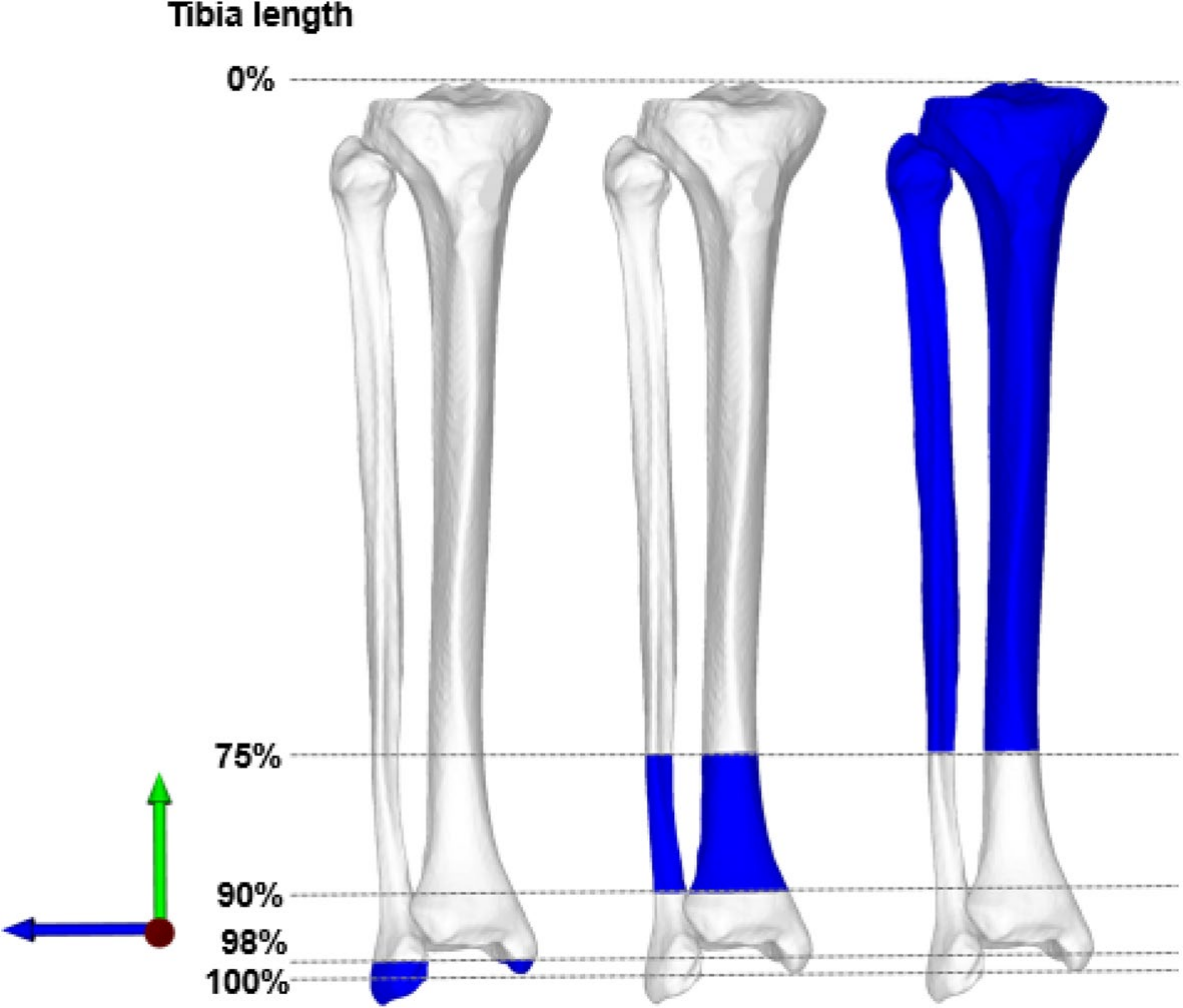
Definition of segments for ankle joint line approximation. Three anatomical segments (blue) were defined for registration and depicted from left to right. Distal (left model): defined at 98% of the tibia length and the corresponding fibula. Middle (center model): Defined from 75–90% of the tibia length and corresponding fibula. Proximal (right model): The segment was defined from 0 to 75% of the tibia length and the corresponding fibula. Coordinate system depicted: z-axis (green arrow), x-axis (blue arrow), y-axis: (red arrow facing the reader)

High-resolution computed tomography (CT) was acquired using a Somatom Definition Flash CT scanner (Siemens ®, Erlangen, Germany) with a slice thickness ranging from 0.5 to 0.6 mm. 3D triangular surface models of 96 paired (48 left, 48 right) tibiae and fibulae were created with manual threshold segmentation and region growing using MIMICS software (MIMICS Medical, Materialise NV, Leuven, Belgium) and imported into the in-house surgical planning software CASPA (Balgrist, University of Zurich). To approximate the original JL from the mirrored contralateral side, an iterative closest point (ICP) algorithm [1] was applied to superimpose the mirrored contralateral model onto the original model, as described in previous studies [9, 24]. A 3D coordinate system was defined according to the International Society of Biomechanics (ISB); [7] z-axis: defined as the anatomical tibia axis defined by an oriented bounding box (OBB), [9] x-axis: directing lateral towards the fibula, y-axis: directing anterior (Fig. 1).

### Definition of tibia and fibula segments for contralateral registration

As segment selection and included anatomical structures potentially improve the accuracy to approximate the original model [24], we defined three distinct segments of the lower leg to restore the JL, excluding the potentially deformed distal tibial plafond. The contralateral lower leg model was mirrored and three anatomical segments were defined (Fig. 1). Previously, Hodel et al. demonstrated decreased accuracy of JL approximation at the knee, when using the tibia or fibula solely due to reported side-to-side differences [9]. To mitigate this effect, we decided to define three segments consisting of the combined tibia and corresponding fibula. We included previously described anatomical landmarks: the distal fibular tip, anterior and posterior colliculi of the medial malleolus [8].

- *Distal segment*: Including the distal fibular tip, anterior and posterior colliculi of the medial malleolus. The segment was defined at 98% of the tibia length and the corresponding fibula (Fig. 1, left).
- *Middle segment:* Defined from 75–90% of the tibia length and corresponding fibula. (Fig. 1, middle).
- *Proximal segment: The* segment was defined from 0 to 75% of the tibia length and the corresponding fibula and can be used in case of severe bone loss, posttraumatic deformity or previous supramalleolar osteotomy (Fig. 1, right).

The surface registration algorithm to superimpose the mirrored contralateral models onto the original model was repeated for all three segments of the tibia and fibula of predefined lengths, as described before.

### Definition of joint line and accuracy of joint line restoration

**Fig. 2:**
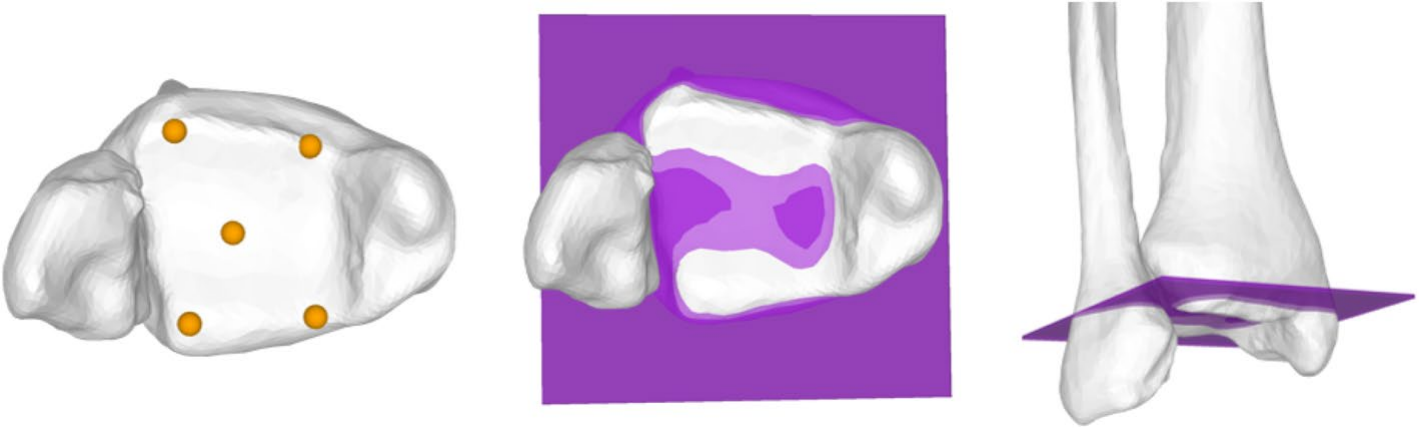
Definition of the ankle joint line. The average of five standardized surface landmark points (two at the anterior ridge, two at the posterior ridge, one centered at tibial plafond) define the ankle joint line

The ankle JL was defined as the average plane of five surface registration points on the tibial plafond in a standardized fashion (two at the anterior ridge, two at the posterior ridge and one at the center) (Fig. 2) as described by Hodel et al. [10] The approximation of the JL based from the contralateral side was measured in mm in direction of the anatomical tibia axis (z-axis) (positive values indicating an elevation of the JL, negative values indicating a distalisation). In addition, the mean absolute error of the approximated JL compared to the original JL was calculated for each segment and is subsequently referred to as JL error.

### Validity testing of the joint line definition

To assess the validity of the novel described JL definition, two independent observers defined the JL in 10 randomly selected ankles. The mean absolute error of the JL definitions between the observers was reported.

### Side-to-side differences of anatomical landmarks with respect to the ankle joint line

**Fig. 3:**
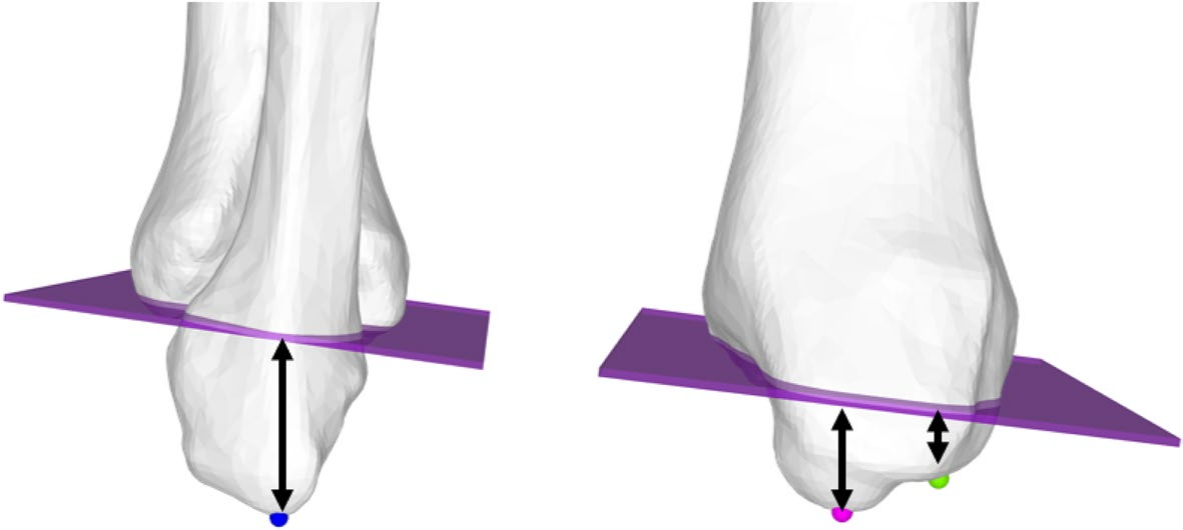
Fibular tip and medial malleolar anterior and posterior colliculus distance to ankle joint line. Left: lateral view depicts fibular tip (blue sphere). Right: medial view depicts anterior medial colliculus (pink sphere) and posterior medial colliculus (green sphere). Shortest distance of anatomical landmarks to ankle joint line is measured (mm, black arrows)

**Fig. 4:**
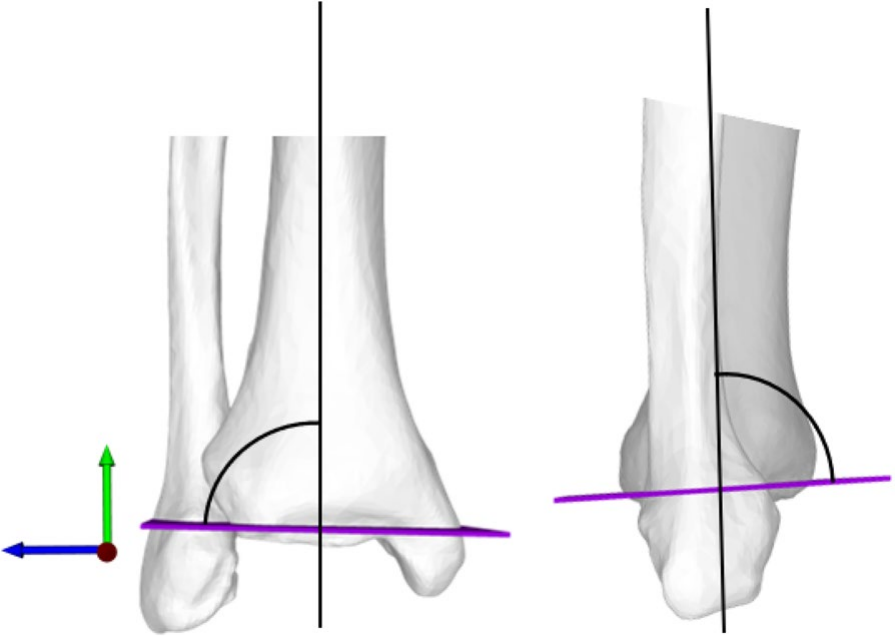
Lateral distal tibial angle (LDTA) and anterior distal tibial angle (ADTA) and Left: LDTA measured as projected 2D angle in frontal plane (plane normal = red arrow). ADTA measured as projected 2D angle in sagittal plane (plane normal = blue arrow)

The length of the tibia and fibula model were defined by the OBB [19]. Side-to-side differences are reported as mean absolute values. The closest distance of the distal fibular tip, anterior, and posterior medial colliculi to the JL were measured using an automatic surface registration sphere on the most distal point of the fibular tip, and anterior and posterior medial malleolar colliculi (Fig. 3). Additionally, the anterior distal tibial angle (ADTA) and lateral distal tibial angle (LDTA) were measured as projected 2D angles in the frontal plane (plane normal = y-axis) and sagittal plane (plane normal = x-axis) respectively (Fig. 4). All measurements were performed by two readers in 40 lower legs to assess inter-reader reliability.

### Statistics

Normal distribution of the data was tested with Shapiro–Wilk’s test and confirmed using histograms. Data are reported as mean ± standard deviation and range. Friedman's test was performed to analyze differences of ankle JL approximations and JL error among the three segments including post-hoc Dunn-Bonferroni testing. Gender, age, height, weight and side-to-side differences of the tibia length, fibula length, distal fibular tip, and medial colliculi to JL distance were included in a linear regression model to analyze their influence on JL error. Side-to-side differences were analyzed using paired t-tests or Wilcoxon's rank-sum test as appropriate. Absolute side-to-side differences between the anatomical landmarks were compared using Friedman's test and post-hoc Dunn-Bonferroni testing. Inter-reader reliability was calculated using intraclass correlation coefficients (ICC) assuming a two-way mixed-effect and absolute agreement and graded according to Fleiss et al. (> 0.75 indicating excellent reliability) [6]. The significance was set < 0.05. Data were analyzed with SPSS version 26 (SPSS Inc, Chicago, IL, USA).

## Results

### Validity testing of the joint line definition

The JL definition demonstrated a mean error of 0.3 ± 0.2 mm (range: 0.1 to 0.6 mm) between the two observers.

### Accuracy of joint line restoration

**Fig. 5:**
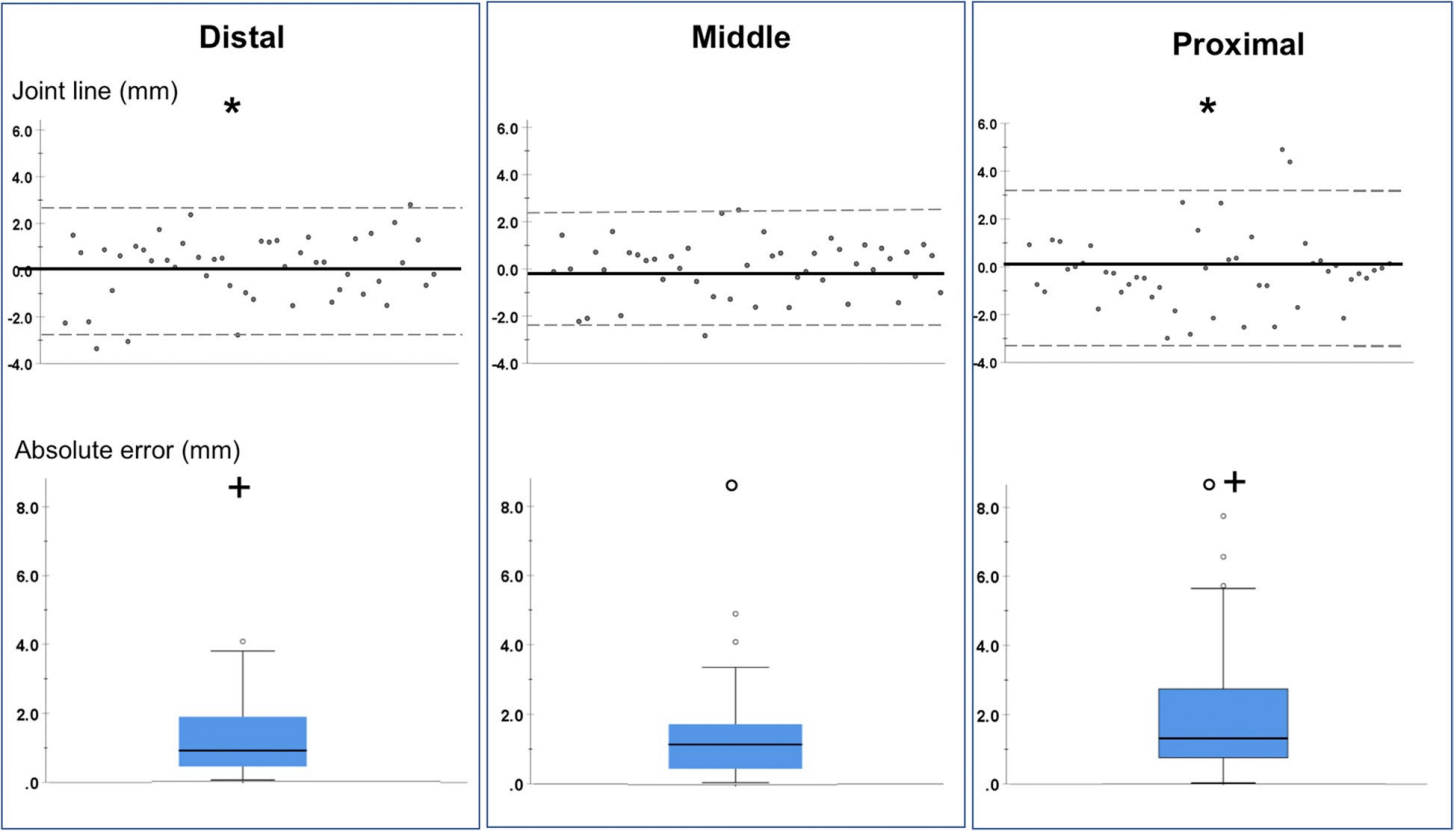
Joint line approximation and absolute error among the distal, middle, and proximal segment

Mean JL approximation was 0.1 ± 1.4 mm (range: -3.4 to 2.8 mm) for the distal, 0.1 ± 1.2 mm (range: -2.8 to 2.5 mm) for the middle and -0.2 ± 1.6 (range: -3.0 to 4.9 mm) for the proximal segment (*p* = 0.007) (Fig. 5).

The JL error was highest for the proximal segment 2.0 ± 1.7 mm (range: 0 to 7.7 mm) and decreased for the middle segment 1.3 ± 1.0 mm (range: 0 to 4.9 mm) and for the distal segment 1.2 ± 1.0 (range: 0 to 4.0 mm) (*p* = 0.003) (Fig. 5).

In multiple regression analysis no significant influence of gender, age, height, weight and side-to-side differences of the tibia length, fibula length, distal fibular tip, and medial colliculi on JL error of each segment could be demonstrated (n.s.).

### Side-to-side differences of anatomical landmarks with respect to the ankle joint line

No significant side-to-side differences could be demonstrated for the distances of the fibular tip, anterior and posterior medial colliculi to the JL, ADTA and LDTA. All measurements demonstrated good to excellent inter-reader agreement. (all summarized Table 1).

**Table 1:** Side-to-side differences of anatomical landmarks with respect to the ankle joint line

	**Left**	**Right**	**Absolute side-to-side difference**	***p*-value**	**ICC (95% CI; *p*-value)**
Fibular tip to JL (mm)	22.0 ± 3.7 (14.1 to 29.1)	21.9 ± 3.9 (14.7 to 28.8)	1.4 ± 1.1 (0 to 5.9)	0.702	0.96 (0.92–0.98; *p* < 0.001)
Anterior medial colliculus to JL (mm)	12.7 ± 1.9 (7.9 to 15.7)	12.9 ± 1.9 (6.9 to 16.3)	0.8 ± 0.7 (0 to 3.0)	0.390	0.78 (0.56–0.89; *p* < 0.001)
Posterior medial colliculus to JL (mm)	7.5 ± 1.8 (3.8 to 10.8)	7.8 ± 2.0 (2.5 to 12.5)	0.8 ± 0.8 (0 to 4.3)	0.079	0.87 (0.74–0.94; *p* < 0.001)
ADTA (°)	82.8 ± 2.3 (78.6 to 88.7)	82.6 ± 2.6 (77.8 to 89.7)	1.6 ± 1.1 (0 to 5.6)	0.773	0.72 (0.47–0.85; *p* < 0.001)
LDTA (°)	87.7 ± 2.0 (80.8 to 89.9)	87.3 ± 2.4 (81.1 to 89.9)	1.7 ± 1.4 (0 to 6.0)	0.238*	0.79 (0.60–0.89; *p* < 0.001)

Mean ± standard deviation (range) are reported. *Wilcoxon's rank sum-test, remaining paired t-test

*JL* Joint line, *ADTA* Anterior distal tibia angle, *LDTA* Lateral distal tibia angle, *ICC* Intraclass correlation coefficient, *CI* Confidence interval

## Discussion

The most important finding of this study is that the contralateral tibia and fibula can reliably be used to restore the original ankle JL. The inclusion of the medial or distal segment of the tibia and fibula improved the accuracy of JL restoration compared to the proximal segment.

To our knowledge, there is only one study assessing the ankle JL level before and after TAA. The authors measured the JL level on weight-bearing radiographs using the joint line height ratio and found an elevated JL in end-stage ankle osteoarthritis [19]. Postoperatively the JL elevation remained. The disadvantage of this method is that it can no longer be used if the anatomical landmarks are altered preoperatively or postoperatively (e.g., malleolar osteotomy) or if posttraumatic degeneration such as a malunion is present. The advantage of the segments used in the present study is that proximal bony parts were included in addition to the distal anatomical landmarks, which could be used in post-traumatic deformities. The increased accuracy for the middle and distal segment may be explained by the inclusion of distinct anatomical landmarks as the distal fibular tip, the fibular notch and the anterior and posterior medial colliculi. Furthermore, we demonstrated that in addition to the contralateral distal fibular tip or the anterior and posterior medial colliculi, the ADTA and LDTA can be reliably used for planning a revision TAA, with only minor side-to-side differences. The accuracy with which the JL of the distal ankle must be restored is unclear, especially in view of the fact that to date the influence on clinical and functional outcome is unknown. The reported accuracy of the JL error of 1.2 mm is within the range of surgical precision as reported in TKA with the use of robotic assistance [22]. Therefore, the reported accuracy is deemed acceptable for clinical applicability in our point of view.

To allow a full ROM and well-balanced TAA, surgical release techniques around the ankle joint have been suggested [15]. However, the influence of JL elevation or distalisation on ligament and tendon lengthening, potentially resulting in impaired function and decreased ROM, has not been investigated yet. Nevertheless, it seems logical that elevation or distalisation of the JL alters the lever arm of peri-articular ligaments and tendons, which may negatively affect muscle function and ankle stability. As previously described in total knee arthroplasty, it is known that a change in JL level is associated with limited range of motion (ROM), joint stiffness, pseudo-patella baja, and poor clinical outcome [2, 20]. Further studies investigating the relationship between the ankle JL level and the clinical and patient-reported outcome in foot and ankle surgery are needed to provide more accurate statements in the future.

The described side-to-side differences of the ADTA and LDTA can be used to guide frontal and sagittal alignment when planning TAA. Especially, when extensive bone loss or ligament instability is present, the use of the contralateral anatomy is of great help. This is of particular interest in a revision setting. Restoring frontal and sagittal alignment resulting in a well-balanced, congruent tibiotalar articulation is proposed by previous authors [13, 26], as restoration of a neutral alignment in TAA has been reported with good clinical outcome [16]. However, the extent to which a mechanical alignment should be targeted remains debatable as good clinical outcome at mid-term follow-up has been described with mild component malalignment [3]. This raises questions if a kinematic alignment could improve functional outcome compared to a strict correction of the mechanical alignment. This is an ongoing discussion in TKA for example. In recent years, the focus shifted towards restoring the original anatomy using customized prostheses, with the overall goal of improving the functional outcome by mimicking the native kinematic behavior and restore ligament balancing [14]. This concept may also play a role for the ankle joint in the future. Furthermore, the reported findings are of clinical relevance when addressing post-traumatic deformities of the ankle. The restoration of the JL and the anatomical frontal and sagittal JL alignment (ADTA, LDTA) can be reconstructed with the help of the contralateral anatomy.

The main limitation of the present study is that the 3D registration method depends on a healthy contralateral anatomy, and therefore preoperative assessment is only possible in healthy bone. To mitigate this limitation, three different anatomical regions were selected and analyzed to allow a registration despite the presence of a deformity, osteoarthritis, or previous surgery at a certain segment of the contralateral side. Finally, information on the medical history of the cadavers was limited. For this reason, specimens with evidence of deformity, previous surgery, or fracture were excluded.

## Conclusion

3D registration of the contralateral tibia and fibula reliably approximated the original ankle JL. The contralateral distal fibular tip, anterior and posterior medial colliculi, ADTA and LDTA can be used reliably for the planning of revision TAA with small side-to-side differences reported.

## Data Availability

The datasets used and/or analysed during the current study are available from the corresponding author on reasonable request.
